# A Real-World Experience of Clinical, Biochemical and Genetic Assessment of Patients with Homozygous Familial Hypercholesterolemia

**DOI:** 10.3390/jcm9010219

**Published:** 2020-01-14

**Authors:** Maria Donata Di Taranto, Carola Giacobbe, Alessio Buonaiuto, Ilenia Calcaterra, Daniela Palma, Giovanna Maione, Gabriella Iannuzzo, Matteo Nicola Dario Di Minno, Paolo Rubba, Giuliana Fortunato

**Affiliations:** 1 Dipartimento di Medicina Molecolare e Biotecnologie Mediche, Università degli Studi di Napoli Federico II, 80131 Naples, Italy; car.giacobbe@gmail.com (C.G.); palmad@ceinge.unina.it (D.P.); maione@ceinge.unina.it (G.M.); 2 CEINGE S.C.a r.l. Biotecnologie Avanzate, 80131 Naples, Italy; 3 Dipartimento di Medicina Clinica e Chirurgia, Università degli Studi di Napoli Federico II, 80131 Naples, Italy; ale.buonaiuto@gmail.com (A.B.); ileniacalcaterra@hotmail.it (I.C.); gabriella.iannuzzo@unina.it (G.I.); rubba@unina.it (P.R.); 4 Dipartimento di Scienze Mediche Traslazionali, Università degli Studi di Napoli Federico II, 80131 Naples, Italy; dario.diminno@hotmail.it

**Keywords:** homozygous familial hypercholesterolemia (HoFH), *LDLR* pathogenic variants, genetic screening, LDL-cholesterol, coronary heart disease, familial hypercholesterolemia prevalence

## Abstract

Homozygous familial hypercholesterolemia (HoFH), the severest form of familial hypercholesterolemia (FH), is characterized by very high LDL-cholesterol levels and a high frequency of coronary heart disease. The disease is caused by the presence of either a pathogenic variant at homozygous status or of two pathogenic variants at compound heterozygous status in the *LDLR*, *APOB*, *PCSK9* genes. We retrospectively analyzed data of 23 HoFH patients (four children and 19 adults) identified during the genetic screening of 724 FH patients. Genetic screening was performed by sequencing FH causative genes and identifying large rearrangements of *LDLR*. Among the HoFH patients, four out of 23 (17.4%) were true homozygotes, whereas 19 out of 23 (82.6%) were compound heterozygotes for variants in the *LDLR* gene. Basal LDL-cholesterol was 12.9 ± 2.9 mmol/L. LDL-cholesterol levels decreased to 7.2 ± 1.8 mmol/L when treated with statin/ezetimibe and to 5.1 ± 3.1 mmol/L with anti-*PCSK9* antibodies. Homozygous patients showed higher basal LDL-cholesterol and a poorer response to therapy compared with compound heterozygotes. Since 19 unrelated patients were identified in the Campania region (6,000,000 inhabitants) in southern Italy, the regional prevalence of HoFH was estimated to be at least 1:320,000. In conclusion, our results revealed a worse phenotype for homozygotes compared with compound heterozygotes, thereby highlighting the role of genetic screening in differentiating one genetic status from the other.

## 1. Introduction

Familial hypercholesterolemia (FH) is an inherited disease characterized by high levels of LDL-cholesterol (LDL-c) which leads to premature coronary heart disease (CHD). Other clinical signs include tendon xanthomas and corneal arcus caused by cholesterol accumulation in tissues [1]. Genetic defects causing the disease are pathogenic variants present in genes encoding proteins related to LDL particle uptake, i.e., genes encoding the LDL receptor (*LDLR*), the apolipoprotein B (*APOB*), the proprotein convertase subtilisin/kexin type 9 (*PCSK9*) inducing LDLR degradation, and the low-density lipoprotein receptor adapter protein 1 (*LDLRAP1*) mediating the interaction of LDLR with clathrin [2].

Based on the number of altered alleles, two forms of FH are recognizable: Heterozygous (HeFH) and homozygous (HoFH). The latter is the severest form of FH, since LDL-c levels above 13 mmol/L (500 mg/dL) are usually reported as being associated with a high frequency of premature CHD. Untreated HoFH could develop CHD even in childhood [3].

The heterozygous form of FH is the most frequent genetic disease, as its prevalence has been recently re-estimated at about 1:250 [4]. Furthermore, the frequency of HoFH has been estimated at 1:160,000–300,000, which is also much higher than previously estimated [3]. The identification of FH patients is usually hard due to the high variability of its clinical and biochemical phenotypic expression, leading to underdiagnosis and undertreatment of patients [5]. Identifying a dramatic increase of LDL-c may facilitate the detection of HoFH patients whereas, in some cases, the phenotypic overlaps with HeFH patients may undermine clinical differentiation [3]. 

Recently, there has been growing interest about genetic/clinical heterogeneity in relation to the therapeutic response of patients with HoFH [6,7,8,9,10,11]. In order to better characterize the features of HoFH, we collected and retrospectively analyzed the genetic, biochemical, and clinical data of about 23 HoFH patients who attended our lipid clinic over the last 10 years. A follow-up analysis was performed in order to evaluate the patients’ response to therapy. 

## 2. Experimental Section

### 2.1. Study Population, Clinical and Biochemical Assessment

We retrospectively collected and analyzed data about 23 HoFH patients from a population of 724 patients who—from 2008 to 2018—attended the Lipid Clinic of Naples University Hospital, which is the reference Center for Campania Region, in Southern Italy. All patients that resulted HoFH at the genetic screening were selected for the study. Informed consent was obtained for each patient. The study was performed according to the current version of the Helsinki Declaration and then approved by the Ethical Committee of the “Università degli Studi di Napoli-Federico II” (Number 262/17, 29 November 2017).

The presence of hypercholesterolemia, tendon xanthomas and premature CHD (in the patient), family history of hypercholesterolemia, xanthomas and family history of premature CHD are the main features which, according to the Dutch Lipid Clinic Network (DLCN) and Simon Broome criteria, lead to FH clinical diagnosis. Patients with a clinical picture and a family history of Familial Combined Hyperlipidemia were excluded [12,13]. Reported data are integrated into LIPIGEN, an Italian registry of patients with familial dyslipidemias [14,15,16].

Body mass index (BMI), used as a measure of general obesity, was calculated as weight (kilograms) divided by height (in m^2^). Total cholesterol, triglycerides, HDL-cholesterol (HDL-c) concentration were measured using standard enzymatic methods in fasting plasma samples. LDL-cholesterol (LDL-c) was calculated according to the Friedewald formula.

### 2.2. Genetic Analysis

Genomic DNA was extracted by Nucleon BACC2 kit (GE Healthcare, Chicago, IL, USA) according to the instructions. Genetic screening was performed by Sanger sequencing of the promoter region, along with all 18 exons and intron-exon junctions of the *LDLR* gene (NM_000527.4). Primers are reported in Supplemental Table S1. The polymerase chain reaction (PCR) amplification was performed using the Promega PCR Master Mix according to the manufacturer’s instructions. Reactions were carried out in 30 μL containing 150 ng of DNA and 15 µmol of each primer. Direct sequencing analysis of the purified PCR product was carried out using the BigDye terminator cycle sequencing ready reaction kit and an ABI Prism 3100 DNA genetic analyzer (Applied Biosystems, Foster City, CA, USA). CodonCode Aligner software was used for sequence analysis. To search for large rearrangements in the *LDLR* gene, the Multiplex ligation dependent probe analysis (MLPA) was performed according to the manufacturer’s instructions. Our standard protocol for genetic diagnosis also included the analysis of *PCSK9* and *APOB* if no *LDLR* variants were identified [17,18]. Since two clearly pathogenic variants in the *LDLR* gene were identified, no further analysis of the *PCSK9* and *APOB* genes was performed. The presence of the variants was ascertained in both parents, confirming that the two variants were present on the two different alleles. Variants were checked against the Human Gene Mutation Database (HGMD). The following databases were used to evaluate the minor allele frequency (MAF): Exome Aggregation Consortium (ExAC), genome Aggregation Database (genomAD), Exome Variant Server (EVS), and 1000 genomes (1kG) and dbSNP 149 (NCBI). Variants were reported according to the Human Genome Variation Society nomenclature. All reported variants were rare and classified either as pathogenic or likely pathogenic according to the guidelines of the American College of Medical Genetics (ACMG) [19]. Some of these variants were also functionally tested confirming the protein defect [20,21].

Nonsense, splicing, or deletion/insertion leading to frameshift and large rearrangements were defined as null variants. 

### 2.3. High-Resolution Carotid Ultrasound

Carotid B-mode ultrasound examinations were performed by a certified sonographer using an ESAOTE AU4. The scanning of the distal 1.0 cm of the near and far walls of the common carotid arteries was carried out using the crest at the origin of the bifurcation as an anatomical landmark to identify this segment. In each examination, the sonographer used different scanning angles (anterior, lateral, and posterior) to allow for the identification of the greatest intima-media thickness (IMT) in each wall. The frame that contained the thickest IMT for each of the four carotid walls was selected. The overall coefficient of reliability was 0.872 for maximum IMT of standard carotid sites. This figure includes instrument, subject, sonographer and reader variabilities [22]. Previous applications of this method were reported in [23,24,25]. In this study, carotid plaque was defined as IMT ≥ 1.2 mm, with loss of parallelism of ultrasound interfaces.

### 2.4. Statistical Analyses 

Statistical analyses were performed using SPSS version 18.0 (SPSS, Inc., Chicago, IL, USA). Continuous variables were described as mean and standard deviation because all had a parametric distribution, as shown by the Kolmogorov–Smirnov test. Mean comparisons were performed by *t*-test. Comparisons between LDL-c levels before and after therapies were assessed by paired *t*-test. Categorical variables were reported as absolute number and percentage. Frequency comparisons were made using a chi-square test. A value of *p* < 0.05 was considered statistically significant.

## 3. Results

### 3.1. Genetic Screening 

The genetic status of the 23 patients and their familial relations are reported in Table 1. All patients reported here carried pathogenic variants in the *LDLR* gene: Four patients (17%) were homozygotes and 19 (83%) were compound heterozygotes.

A total of 23 different pathogenic variants were identified, highlighting the high genetic heterogeneity of FH in this population. Most of the variants were missense changes (17 different variants). One regulatory variant, one nonsense variant, one small deletion, two splicing alterations, and one large duplication were also found. Based on the variant type, five patients carried a null and a defective variant in *LDLR*, whereas 19 patients carried two defective variants. 

All patients but one originated from the Campania region, whereas the remaining patient originated from Jordan and carried a variant in the 5’ UTR region of the *LDLR* gene (c.-156C > T), which has already been identified in Israeli FH patients [32]. The most frequent variants were the p.(Gly592Glu), also known as Foggia-1 or Naples-4, which was identified in nine patients (two homozygotes) and the p.(Cys379Arg), also known as Naples-1, identified in five patients (one homozygote). 

Considering only the 19 unrelated patients from the Campania region which has a population of about six million inhabitants, the calculated prevalence of HoFH was 1:320,000 individuals. However, the presence of additional affected patients cannot be excluded.

### 3.2. Biochemical and Clinical Features

Biochemical characteristics and clinical features of patients are reported in Table 2. Higher levels of basal total cholesterol, LDL-c, and non-HDL cholesterol were observed in homozygous patients compared with compound heterozygous patients. No differences were observed between pediatric and adult patients or between patients carrying a null and a defective variant or two defective variants.

There were 11 out of 23 patients with basal LDL-c ≥ 13 mmol/L (500 mg/dL) and 12 out of 23 with LDL-c lower than this threshold (minimum observed value: 7.7 mmol/L–297 mg/dL).

There were no patients with diabetes mellitus. Tendon xanthomas were present in almost all patients, whereas corneal arcus and premature CHD were present in about half of the patients. It is likely that the low prevalence of CHD was related to the young age of patients. On the other hand, documented familial history highlighted that the presence of hypercholesterolemia was the most prevalent feature among first degree relatives. 

We also calculated DLCN scores for adult patients, both considering only the clinical/biochemical features and including the eight points due to the presence of causative variants. It is noteworthy that the lowest DLCN score in the absence of genetic screening was eight points, indicating that, even in HoFH patients, the phenotypic features did not allow to reach the threshold score for definite FH (nine points).

### 3.3. Management of Hypercholesterolemia

Lipid levels after traditional therapy were available for 22 out of 23 patients, while the data of one pediatric patient undergoing lipoprotein apheresis were missing. Means of lipid profile are reported in Table 2 as “Post-therapy 1” values. No differences of lipid profile after traditional therapy were observed between homozygotes and compound heterozygotes. Overall, traditional therapy was associated with a LDL-c reduction of approximatively 50%. 

**Figure 1:**
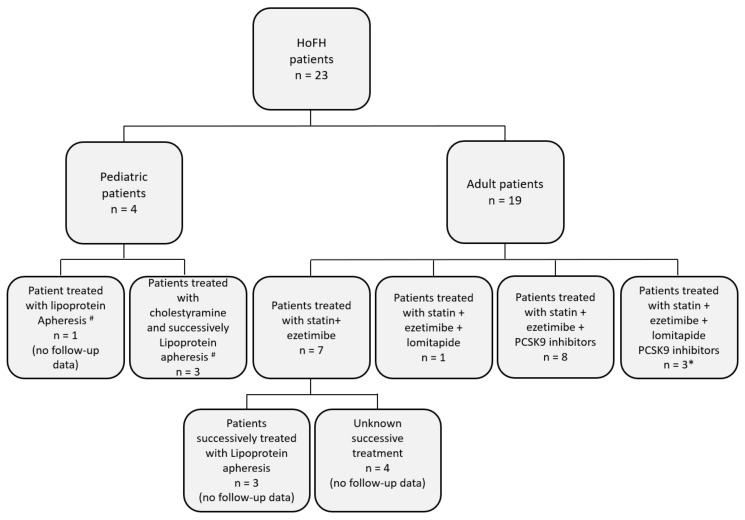
Diagram reporting treatment of Homozygous Familial Hypercholesterolemia patients. The different therapies used in homozygous familial hypercholesterolemia (HoFH) patients are reported. # Lipid profile after lipoprotein apheresis was unavailable; * Patients treated with lomitapide were five, but two suspended the therapy due to diarrhea.

Since the therapeutic target was not reached for any patient, additional therapies were initiated. Therapies followed by patients are detailed in Figure 1. Seven patients were lost at follow-up, and three of the seven were undergoing lipoprotein apheresis. For one patient, lomitapide was added to the treatment with statin and ezetimibe. For 11 patients, treatment was integrated by adding anti-*PCSK9* antibodies, and for three of these patients, lomitapide was also used. Treatment with lomitapide was poorly accepted by patients, and two patients suspended the therapy due to diarrhea.

Lipid values after therapy with anti-PCSK9 antibodies and/or lomitapide are reported in Table 2 as “Post-therapy 2” values. Homozygous patients showed a poorer response in terms of total cholesterol, LDL-c, non-HDL cholesterol and the LDL/HDL cholesterol ratio than compound heterozygous patients. 

**Figure 2:**
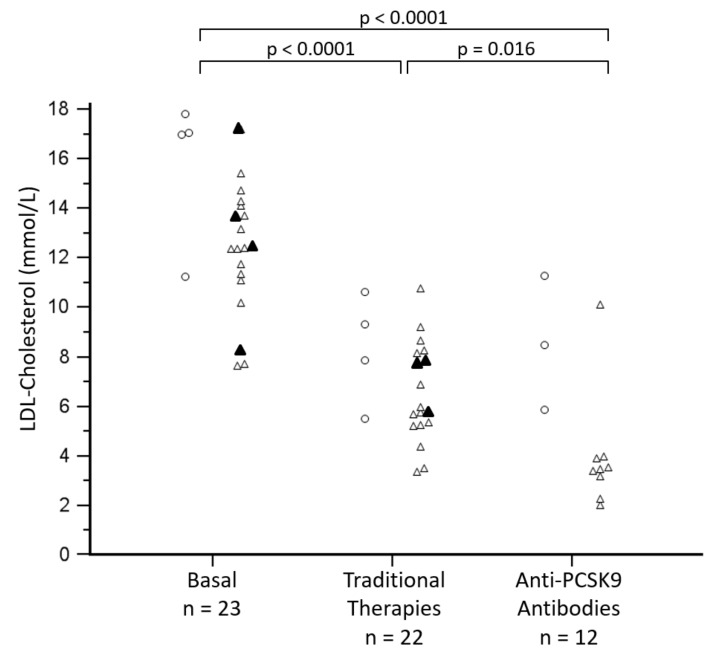
Lipid levels before and after therapy in homozygous patients. LDL-cholesterol levels in mmol/L before therapy, after traditional therapy (statin therapy plus ezetimibe in adults, or cholestyramine in three children) and after innovative therapies (lomitapide or anti-*PCSK9* antibodies) are reported. Open circles indicate the homozygous patients (all adults), whereas triangles indicate compound heterozygous patients (open triangles indicate the adults and black ones indicate the children). Statistical significances were evaluated by paired *t*-test.

Figure 2 reports LDL-c changes during the patient treatment, highlighting that HoFH patients always showed very high LDL-c. Even with innovative therapies none of the patients reached the therapeutic goal. However, LDL-c levels were statistically lowered by both traditional therapies and treatment with anti-*PCSK9* antibodies (Figure 2).

## 4. Discussion

The study of genetically confirmed HoFH patients offered new insights into FH while helping to determine its actual prevalence, which was shown to be higher than previously estimated [35]. In this retrospective study, we reported the data of 23 genetically confirmed HoFH patients. Five patients carried a null variant, i.e., a variant causing a dramatic change in the protein structure so that even if the protein is still present, it is not functional. Despite the predicted worse phenotype of null variant carriers, we did not find any difference between these patients and the carriers of two defective variants. Whereas, among heterozygous FH patients, different phenotypes were observed on the basis of the variant type, being LDL-c in patients carrying a null variant higher than in patients carrying a defective variant [23,36]. However, no patients with two null variants were identified in this study, probably due to the fact that the severe phenotype in these patients are rarely observed [11,37]. 

Higher basal LDL-c was found in homozygous patients (the same variant in both alleles) compared to compound heterozygous patients (two different variants on the two alleles). This difference becomes even more evident after therapy with statin, ezetimibe, and anti-PCSK9 antibodies. Similar data were also reported for a Spanish population of HoFH patients [37]. From this perspective, genetic screening could allow for a better characterization of the response to *PCSK9* inhibitors in HoFH, although larger studies are required.

Genetic screening allows us to unequivocally discriminate between HeFH and HoFH [2]. A correct identification and treatment of HoFH patients is essential to prevent premature CHD and carotid plaque development, whose prevalence was very high among the adult HoFH patients, whereas it is usually lower in heterozygotes [23]. 

The phenotypic heterogeneity observed among HeFH patients was also observed among HoFH patients [11], leading to possible difficulties in patient identification despite the very severe phenotype. According to the guidelines of the European Atherosclerosis Society [3], HoFH should be suspected when LDL-c levels are higher than 13 mmol/L (500 mg/dL). In our study, only 11 out of 23 patients (48%) showed LDL-c levels above this threshold, suggesting that diagnostic criteria should be revised.

HDL-c was very low (1.1 ± 0.2 mmol/L), similar to the levels observed in our previous study [23]. In that study, HDL-c levels resulted statistically lower in HoFH than in HeFH patients. Several evidences of low HDL-c levels were reported in FH and, in particular, in HoFH [38]. Molecular mechanisms underlying decreased HDL-c are related to both a high activity of hepatic lipase [39] and increased ApoA1 catabolism [40]. In this respect, we also calculated the LDL/HDL ratio that, although not different at basal measurements, was about twofold higher in homozygotes than in compound heterozygotes after therapy with anti-PCSK9 antibodies. We previously observed that the LDL/HDL ratio was the best indicator of the presence of a pathogenic variant in a pediatric population with clinical suspect of FH [38].

Among our HoFH patients, we found a high frequency of two variants—p.(Gly592Glu) and p.(Cys379Arg)—suggesting the presence of variant clusters in our region.

Based on the current data about the inhabitants of Campania, we established the frequency of HoFH at 1:320,000. However, since the presence of still undiagnosed HoFH cannot be excluded, the frequency could be even higher. This frequency is similar to that estimated in the Netherlands [35], whereas it is higher than the Spanish prevalence [37]. The screening of the *APOB* and *PCSK9* genes in our population would have probably identified additional HoFH patients, further increasing the disease prevalence. Future applications of next generation sequencing (NGS) allowing the analysis of all FH causative genes will increase the number of HoFH identified patients and, as a result, HoFH prevalence. However, the massive identification of potential pathogenic variants by NGS should always be followed by an accurate pathogenicity evaluation, including—whenever possible—a functional evidence of the molecular defect [41,42].

The optimal treatment of HoFH requires great efforts from patients to accept lipoprotein apheresis or lomitapide assumption. Our paper describes a real-word experience highlighting the treatment difficulties of HoFH patients. Treatment with anti-*PCSK9* antibodies is well-tolerated by patients and therefore widely used among our patients. A gradual decrease of LDL-c was observed with traditional statin/ezetimibe therapy and with anti-*PCSK9* antibodies therapy. Very recent evidence has indicated that HoFH could be treated with antibodies targeting Angiopoietin-like 3 (ANGPTL3) because their action mechanism is independent of LDLR activity [43].

## 5. Limitations

Since this is a retrospective study and genetic screening was performed through traditional sequencing, no variants in the *APOB* and *PCSK9* genes were searched for. We cannot rule out the presence in the above genes of further rare variants with high or mild impacts on lipid phenotype. Unfortunately, we cannot extend the genetic analysis to the other FH-causative genes (*APOB*, *PCSK9* and *LDLRAP1*) because of unavailability of DNA samples.

Since among the whole FH population, the *APOB* and *PCSK9* genes were not analyzed in all patients, the prevalence of molecularly defined HoFH in the Campania region could be even higher than that reported in this study.

In addition, clinical and biochemical data were retrieved retrospectively, and some follow-up data were unavailable. However, this cross-section of HoFH patient data reflects the real-world experience of difficulties in obtaining accurate data for both the patients and their family members.

## 6. Conclusions

Our results revealed that homozygous patients showed worse lipid profile and worse response to lipid-lowering therapy than compound heterozygotes, highlighting the role of genetic screening in the differentiation of the two genetic statuses besides the differentiation between HeFH and HoFH. The prevalence of HoFH in our region was estimated at 1:320,000 accordingly, with recent estimations reported in other European studies.

## Figures and Tables

**Table 1:** Genetics, ages, and familial relations of the 23 patients with homozygous familial hypercholesterolemia.

Patient	Age (Years)	Gender	Age of the First CHD Event (Years)	Genetic Status	*LDLR* Variant (Nucleotide)	LDLR Variant (Protein)	Variant Classification	Variant References
HoFH-1	29	Female	-	Comp. Heter.	c.1135T > C + c.1567G > A	p.(Cys379Arg) + p.(Val523Met)	Defective/Defective	Both variants [20]
HoFH-2 ^a^	53	Male	-	Comp. Heter.	c.1130G > T + c.2476C > A	p.(Cys377Phe) + p.(Pro826Thr)	Defective/Defective	[20]/[26]
HoFH-3	33	Female	30	Comp. Heter.	c.1646G > A + c.1739C > T	p.(Gly549Asp) + p.(Ser580Phe)	Defective/Defective	Both variants [20]
HoFH-4	29	Male	32	Comp. Heter.	c.367T > C + c.1478_1479delCT	p.(Ser123Pro) + p.(Ser493Cysfs*42)	Defective/Null	[20] (functional activity was assayed in cells from this patient)
HoFH-5	46	Male	-	Comp. Heter.	c.304C > T and c.718G > A	p.(Gln102*) and p.(Glu240Lys)	Null/Defective	[27]/[15]
HoFH-6	35	Male	24	Homoz.	c.1135T > C	p.(Cys379Arg)	Defective	[20]
HoFH-7 ^b^	20	Female	-	Homoz.	c.1775G > A	p.(Gly592Glu)	Defective	[20]
HoFH-8 ^b^	22	Male	-	Homoz.	c.1775G > A	p.(Gly592Glu)	Defective	[20]
HoFH-9	48	Female	-	Comp. Heter.	c.727T > C + c.1775G > A	p.(Cys243Arg) + p.(Gly592Glu)	Defective/Defective	[28]/[20]
HoFH-10	55	Female	55	Comp. Heter.	c.974G > A + c.(940 + 1_941-1)_(2311 + 1_2312-1)dup	p.(Cys325Tyr) + p.(Gly314_Gln770dup)	Defective/Null	[20]/[29]
HoFH-11	36	Male	-	Comp. Heter.	c.352G > T + c.1646G > A	p.(Asp118Tyr) + p.(Gly549Asp)	Defective/Defective	[30]/[20]
HoFH-12	49	Female	-	Comp. Heter.	c.323C > T + c.1586 + 1G > A	p.(Thr108Met) + p.(Thr454_Gly529de) and p.(Gly529_Phe530ins22)	Defective/Null	[31]/[30]
HoFH-13	62	Female	61	Comp. Heter.	c.1135T > C + c.1586 + 5G > A	p.(Cys379Arg) + p.(Thr454_Gly529de)	Defective/Null	[20]/[28]
HoFH-14	46	Male	34	Homoz.	c.-156C > T	p.(?)	Defective	[32]
HoFH-15 ^a^	44	Female	-	Comp. Heter.	c.1130G > T + c.2476C > A	p.(Cys377Phe) + p.(Pro826Thr)	Defective/Defective	[20]/[26]
HoFH-16	64	Female	49	Comp. Heter.	c.1775G > A + c.2054C > T	p.(Gly592Glu) + p.(Pro685Leu)	Defective/Defective	[20]/[33]
HoFH-17	28	Female	-	Comp. Heter.	c.1775G > A + c.2054C > T	p.(Gly592Glu) + p.(Pro685Leu)	Defective/Defective	[20]/[33]
HoFH-18	63	Male	32	Comp. Heter.	c.463T > C + c.1567G > A	p.(Cys155Arg) + p.(Val523Met)	Defective/Defective	[34]/[20]
HoFH-19	40	Female	40	Comp. Heter.	c.1567G > A + c.2054C > T	p.(Val523Met) + p.(Pro685Leu)	Defective/Defective	[20]/[33]
HoFH-20 ^c^	7	Female	-	Comp. Heter.	c.407A > T + c.1775G > A	p.(Asp136Val) + p.(Gly592Glu)	Defective/Defective	[20] (functional activity was assayed in cells from this patient)
HoFH-21	10	Male	-	Comp. Heter.	c.1135T > C + c.1775G > A	p.(Cys379Arg) + p.(Gly592Glu)	Defective/Defective	Both variants [20]
HoFH-22 ^c^	4	Male	-	Comp. Heter.	c.1135T > C + c.1775G > A	p.(Cys379Arg) + p.(Gly592Glu)	Defective/Defective	Both variants [20]
HoFH-23	9	Male	-	Comp. Heter.	c.1739C > T + c.1775G > A	p.(Ser580Phe) + p.(Gly592Glu)	Defective/Defective	[20] (functional activity was assayed in cells from this patient)

HoFH: Homozygous familial hypercholesterolemia; CHD: Coronary heart disease; *LDLR*: LDL receptor; ^a^ siblings; ^b^ siblings; ^c^ cousins.

**Table 2:** Biochemical characteristics and clinical features of all patients, homozygous patients and compound heterozygous patients.

Parameter	Total *n* = 23	Homozygotes *n* = 4	Compound Heterozygotes *n* = 19	Statistical Significance
Age at genetic diagnosis, years	36.2 ± 18.2	30.7 ± 12.1	37.3 ± 19.3	ns
Pre-therapy Total cholesterol, mmol/L	15.2 ± 2.8	18.9 ± 0.4	14.4 ± 2.5	*p* = 0.009
Pre-therapy LDL-cholesterol, mmol/L	12.9 ± 2.9	15.8 ± 3.0	12.3 ± 2.5	*p* = 0.026
Pre-therapy HDL-cholesterol, mmol/L	1.1 ± 0.2	1.1 ± 0.1	1.1 ± 0.2	ns
Pre-therapy Non-HDL cholesterol, mmol/L	14.1 ± 2.9	17.7 ± 0.4	13.4 ± 2.5	*p =* 0.011
Pre-therapy LDL/HDL cholesterol ratio	13.9 ± 5.7	15.4 ± 0.6	13.6 ± 6.3	ns
Pre-therapy Triglycerides, mmol/L	1.2 ± 0.6	1.0 ± 0.1	1.3 ± 0.6	ns
Post-therapy 1 Total cholesterol, mmol/L	8.8 ± 2.0	9.8 ± 2.4	8.2 ± 2.2	ns
Post-therapy 1 LDL-cholesterol, mmol/L	7.2 ± 1.8	8.3 ± 2.2	6.6 ± 2.0	ns
Post-therapy 1 HDL-cholesterol, mmol/L	1.1 ± 0.2	1.1 ± 0.1	1.1 ± 0.2	ns
Post-therapy 1 Non-HDL cholesterol, mmol/L	7.7 ± 2.0	8.7 ± 2.3	7.1 ± 2.2	ns
Post-therapy 1 LDL/HDL cholesterol ratio	6.8 ± 2.2	7.7 ± 1.4	6.2 ± 2.0	ns
Post-therapy 1 Triglycerides, mmol/L	1.1 ± 0.7	0.9 ± 0.4	1.1 ± 0.7	ns
Post-therapy 2 Total cholesterol, mmol/L	6.8 ± 3.1	10.1 ± 2.9	5.7 ± 2.4	*p* = 0.025
Post-therapy 2 LDL-cholesterol, mmol/L	5.1 ± 3.1	8.6 ± 2.7	4.0 ± 2.4	*p =* 0.020
Post-therapy 2 HDL-cholesterol, mmol/L	1.2 ± 0.2	1.1 ± 0.2	1.2 ± 0.2	ns
Post-therapy 2 Non-HDL cholesterol, mmol/L	5.6 ± 3.1	8.9 ± 2.8	4.5 ± 2.4	*p =* 0.023
Post-therapy 2 LDL/HDL cholesterol ratio	4.2 ± 2.6	7.4 ± 1.7	3.3 ± 2.1	*p =* 0.012
Post-therapy 2 Triglycerides, mmol/L	1.0 ± 0.5	0.8 ± 0.2	1.1 ± 0.6	ns
Tendon xanthomas, *n* (%)	19/23 (82.6%)	4/4 (100%)	15/19 (78.9%)	ns
Corneal arcus, *n* (%)	11/23 (47.8%)	3/4 (75.0%)	8/19 (42.1%)	ns
Premature coronary heart disease *, *n* (%)	9/19 (47.4%)	2/4 (50%)	7/15 (46.7%)	ns
Premature cerebral or peripheral vascular disease *, *n* (%)	3/19 (15.8%)	1/4 (25.0%)	2/15 (13.3%)	ns
Presence of carotid plaque *, *n* (%)	13/19 (68.4%)	2/4 (50.0%)	11/15 (73.3%)	ns
First degree with Tendon xanthoma and/or Corneal arcus, *n* (%)	7/23 (30.4%)	4/4 (100%)	3/19 (15.8%)	*p =* 0.001
First degree relative with premature coronary heart disease, *n* (%)	13/23 (56.5%)	2/4 (50.0%)	11/19 (57.9%)	ns
First degree with LDL-cholesterol higher than 4.9 mmol/L, *n* (%)	18/23 (78.3%)	4/4 (100%)	14/19 (73.7%)	ns
Minor relatives with LDL-cholesterol higher than 4.1 mmol/L, *n* (%)	9/23 (39.1%)	4/4 (100%)	5/19 (26.3%)	*p =* 0.006
BMI, kg/m^2^	25.4 ± 5.2	22.9 ± 2.1	26.1 ± 5.7	ns
DLCN score (without genetics) *	15.6 ± 2.6 range 8–18	17.0 ± 1.2 range 16–18	15.2 ± 2.8 range 8–18	ns
DLCN score (with genetics) *	23.6 ± 2.6 range 16–26	25.0 ± 1.2 range 24–26	23.2 ± 2.8 range 16–26	ns

* Premature coronary heart disease, premature cerebral or peripheral vascular disease, and presence of carotid plaque and DLCN scores were reported only for 19 adult patients (four homozygotes and 15 compound heterozygotes); ns: not significant; BMI: Body Mass Index; DLCN: Dutch Lipid Clinic Network.

## Supplementary Materials

The following are available online at https://www.mdpi.com/2077-0383/9/1/219/s1, Table S1: Primers used for direct sequencing of *LDLR* gene.
