# Supplementary material for: A Real-World Experience of Clinical, Biochemical and Genetic Assessment of Patients with Homozygous Familial Hypercholesterolemia
Source: J Clin Med. 2020 Jan 14;9(1):219. doi: 10.3390/jcm9010219 (PMC7019873; doi:10.3390/jcm9010219)
Supplement: Supplementary file 1 [file jcm-09-00219-s001.pdf]

Supplemental Table 1. Primers used for direct sequencing of *LDLR* gene

Amplicon	Primer sequence
Promoter and exon 1 - Forward	GGGCGTCAGCTCTTCACCG
Promoter and exon 1 - Reverse	CAGACTCCTCCCGACCCTCG
Exon 2 - Forward	GGGTTCCTTCTTTGTGTCCTCCA
Exon 2 - Reverse	TTTCCAGCCGCCATCATCAA
Exon 3 - Forward	CAGTGGGTCTTTCCTTTGAGTGACA
Exon 3 - Reverse	GGCCTTGCTCCTTGGCAGAA
Exon 4 - Forward	GCACCGAGAGGGCAGTGGTT
Exon 4 - Reverse	GGAGCCCAGGGACAGGTGAT
Exon 5 - Forward	CTCCCAAAGTGCTGGGATTC
Exon 5 - Reverse	GCGTGGTGGTATACGCCTTT
Exon 6 - Forward	TGAGTGCCAAGCAAACTGAGGC
Exon 6 - Reverse	TTGCACAGGGTGGGCAGAGT
Exon 7 - Forward	CGGCGAAGGGATGGGTAGG
Exon 7 - Reverse	TTACGCACCCGGCCTGAAAT
Exon 8 - Forward	CTTGGTTGGGTTCCCGTGGT
Exon 8 - Reverse	GAGCAAACAGCCTCCCGGTC
Exons 9-10 - Forward	TCTGGGTGCCTCCTCTGGCT
Exons 9-10 - Forward	CCATTCCCTCTGCAGCCCTC
Exon 11 - Forward	CAAGCCACATTTGGAGTTTGGG
Exon 11 - Reverse	CCTTCAGGGAGCAGCTTGGG
Exon 12 - Forward	CCATTGGAGAGGGCGTCACA
Exon 12 - Reverse	TGCGTTCATCTTGGCTTGAGTG
Exons 13-14 -Forward	TGGAGAGAGGGTGGCCTGT
Exons 13-14 -Reverse	CGACCTTGAGGTACCCATTTGACA
Exon 15 - Forward	CAGGCACGTGGCACTCAGAA
Exon 15 - Reverse	GCTGAGGCAACGAATGTGCC
Exon 16 - Forward	CACAAATAAGCCCGTGTGGC
Exon 16 - Reverse	GGATGAGCATGGGTGAGGCA
Exon 17 - Forward	TTTCACTCCAGCCACGGAGC
Exon 17 - Reverse	ACCACCAAGGCCATTGTCCG
Exon 18 - Forward	AGAGGGGTAAAAAGGCAGGA
Exon 18 - Reverse	CAAGCCATTGCATGGGCACT
